# Identifying the active ingredients and contextual factors of social prescribing when used to support the mental health of children and young people: a qualitative study

**DOI:** 10.1007/s00787-025-02734-5

**Published:** 2025-05-17

**Authors:** Mariana Willmersdorf Steffen, Aisling J. Murray, Imaan Mohammed, Francois van Loggerenberg, Paul Heritage, Jennifer Y. F. Lau

**Affiliations:** 1 Youth Resilience Unit, Wolfson Institute of Population Health, Queen Mary University of London, London, UK; 2 Psychology Department, Royal Holloway University of London, London, UK; 3 People’s Palace Projects, School of the Arts, Queen Mary University of London, London, UK

**Keywords:** Social prescribing, Children, Young people, Mental health, Active ingredients

## Abstract

Recent years have seen a rise in mental health challenges among children and young people (CYP), while frontline treatments remain limited. Social prescribing (SP), a holistic approach using community resources to empower individuals in managing their health, offers a potentially more sustainable and acceptable option for supporting CYP’s mental health. Although early evidence is promising, little is known about how SP works. Following the UK Medical Research Council (MRC) framework for developing and evaluating complex interventions (Skivington et al., 2021), this study explores the active ingredients and contextual factors of SP in supporting CYP mental health. Nineteen professionals involved in delivering SP to CYP with mental health difficulties were interviewed. Through template analysis, four active ingredients were identified: (1) SP offers a holistic approach that involves tailoring programmes to a young person’s needs, interests and their readiness, (2) SP offers young people an opportunity to exercise agency in defining their care pathway and engaging in their recovery journey, (3) The development of a professional but unpressured supportive relationship with a skilled adult is an important factor in SP, and (4) SP offers a safe space for young people to discuss their emotional needs and health. Additionally, four contextual factors influencing SP delivery were identified: (1) Supportive organisational environments, (2) Parental buy-in, (3) Public awareness, and (4) Barriers to access. Defining these active ingredients and contextual factors can help consolidate SP for CYP’s mental health, contributing to its wider adoption and impact.

**Supplementary Information:**

The online version contains supplementary material available at 10.1007/s00787-025-02734-5.

## Background

Recent years have seen an unprecedented rise in mental health difficulties among children and young people (CYP) [1]. With demand for services exceeding availability, CYP can face waiting lists of over two years [2]. While frontline treatments for many common mental health difficulties are psychological, targeting cognitive and behavioural factors, their high costs limit accessibility [1]. There is an urgent need for other scalable interventions. Social prescribing (SP) is a way through which trusted individuals, based either at healthcare or community settings, can connect people with non-medical, health-related needs to pre-existing community resources [3]. These consist of non-clinical sources of support, such as youth clubs, gardening groups and arts organisations, which are recommended according to the person’s needs and interests [4]. SP takes a holistic perspective as it considers health and wellbeing as being influenced by a broader range of environmental factors including social and relational aspects [3]. While these features reflect a conceptual definition of SP, the way that it is delivered can vary across models [5]. Both the referrer (usually, but not always, a healthcare professional) as well as the “implementer” (sometimes, a link worker, other times an employee of a third sector organisation or even a “hub” or panel) can vary. The degree to which individuals in these prescribing roles signpost or active facilitate, the extent to which they monitor and follow-up on compliance and their levels of training also vary. Nonetheless, across models, SP is designed to empower people to take control and responsibility of their own health problems [6]. As such, it is a potentially more sustainable and acceptable approach originating in the United Kingdom (UK) [7] that has been steadily growing in Europe [8].

SP has been used to address a range of health outcomes, including mental health [9, 10]. Reviews generally find that SP enhances individuals’ overall health and wellbeing, reducing social isolation [11], loneliness [12] and improving mental health [13]. For CYP, SP has been used to support neurodisability [14] and mental health conditions [15], but this evidence base remains small. A recent rapid evidence review [15] found four papers reporting on improvements to mental health outcomes. These identified increases in personal and mental wellbeing [16–18], goal-based outcomes [18], and self-esteem and confidence [19], as well as reductions in loneliness and perceived levels of support needed [17].

Despite early findings, there is limited understanding of why SP may be effective in addressing CYP’s mental health. With the significant expansion of SP across different countries [8], understanding how these interventions work is crucial to ensure they benefit CYP and have broad reach. In complex interventions like SP, where multiple components contribute to non-linear pathways of change [20], it is important to identify which components drive outcome changes—commonly referred to as active ingredients (AIs) [21]. By examining active ingredients together with their contextual influences, SP interventions can be better designed to support the mental health needs of CYP [22].

A study by Hayes and colleagues [23] explored the barriers and facilitators for implementing SP to support CYP’s mental health. Interpersonal skills related to empathy, listening and motivation, and having a knowledge of CYP, were highlighted as key facilitators for the delivery of SP when supporting CYP’s mental health [23]. Regarding barriers, the authors report on the challenges of working with parents/guardians, as well as on the need for effective supervision, clearer policies and procedures, better training, and access to appropriate resources to engage with CYP effectively [23].

Although informative to the implementation of interventions, Hayes and colleagues [23] did not directly measure AIs or contextual factors. Here, in line with UK Medical Research Council (MRC) framework for developing and evaluating complex interventions [24], we sought to address two research questions: (1) What are the AIs for SP when used to support the mental health of CYP? (2) What contextual factors affect the effectiveness of SP with this population?

## Methods

We adopted a qualitative research design consisting of semi-structured interviews with professionals involved in delivering SP for CYP’s mental health. Ethics approval was granted by Queen Mary Ethics Committee on 14th December 2022 (reference QMERC22.354).

### Recruitment and sampling strategy

Participants were recruited through a SP programme in East London, UK (IM is a previous collaborator of the programme and co-author on this paper) and also through advertising via UK networks/organisations: Social Prescribing Network, the National Academy of Social Prescribing, Active Partnerships, and London Arts and Health. These networks shared the study advertisement through newsletters, internal communications, and social media. Additionally, some interviewees promoted the study to colleagues after their own participation.

### Participants

Professionals were eligible to take part in the study if they were: aged 18 years or over, working in an organisation or service involved in delivering SP for CYP with mental health difficulties, having experience of working directly with at least two clients aged 8–17 years in a SP programme and able to give informed consent. The client age range was determined considering that from around age 8 children begin learning coping mechanisms and emotional regulation strategies; social connections outside of their family also become more salient at this age [25]. These developmental differences may then affect the AIs of interventions such as SP. The upper age limit of 18 was selected as in the UK, it is the age they begin to access adult services.

### Procedure

After obtaining informed consent, participants attended a semi-structured interview, conducted by the lead researcher (MS). They first completed a sociodemographic form, providing their age, gender, ethnicity, education, years of experience in the current role and in working with CYP. Interviews followed a topic guide with open-ended questions about the benefits, accessibility and challenges of SP for CYP’s mental health. Participants were also asked about their professional experiences in these schemes, particularly regarding training opportunities and support available when/if facing distressing events.

Interviews continued until data saturation was reached, at which point no new information or themes relevant to the analysis emerged from participant responses [26]. In total, we conducted 18 interviews with 19 participants, two of them were interviewed together upon request. Interviews were conducted in English between January and August 2023, lasted 55 min on average, and took place either online using Zoom (*n* = 16) or where possible, in person (*n* = 2). All participants received a £25 voucher in appreciation of their time.

### Data analysis

All interviews were audio recorded, transcribed verbatim using a transcription software (Otter.AI) and corrected against the recording. Transcripts were uploaded to NVivo (version 14.23.4) and analysed using template analysis [27]. This technique was deemed most appropriate as it maintains a degree of objectivity by incorporating a verification step, wherein a second researcher participates in the coding process using a codebook, whilst allowing for researcher reflexivity throughout the analysis [28].

Consistent with this method, a combination of inductive and deductive approaches was used. The deductive approach was used to create predetermined codes and themes guided by notes taken during data collection, consultations with partners, and frameworks and questions from the existing literature. This informed the development of an initial coding framework, which was used to identify data that fitted within a broad SP framework around the core study questions. Concurrently, an inductive approach was employed where unanticipated codes were created through line-by-line coding to capture emerging, unanticipated information expressed by participants which was also relevant to the research questions. The deductive and inductive codes were combined into the emerging themes within the codebook. To develop the initial codebook, the lead researcher (MS) and a second researcher (AM) independently coded two transcripts each and met to discuss and combine codes. In accordance with the quality checks for template analysis [29], all codes generated by each researcher were carefully reviewed and potential main patterns in the data were discussed and documented. A third researcher (FvL) reviewed the initial codes and suggested changes to the codebook. The template was then applied to the remainder of the data, revised and reapplied as researchers met to discuss potential themes and incorporate new codes after subsets of two to three transcripts were coded.

In the critical realist approach adopted, interpreting the data is key to identifying its underlying patterns [30]. Reflexivity is central throughout the research process, as the researcher’s choices shape the depiction of reality. This study adhered to the reflexivity guidelines outlined by Olmos-Vega et al. [31], with the lead researcher engaging in personal, interpersonal, methodological, and contextual reflexivity throughout the research process. Personal reflexivity involved reflecting on her own position, including her interest in working with young people and her choice to explore resource-oriented approaches for supporting their mental health. Interpersonal reflexivity considered how her background and role might influence interactions with participants and the interpretation of their narratives. Methodological reflexivity was central in the choice of template analysis, acknowledging that this structured approach provided the ideal combination of analytical distance and structured reflexivity to answer the research questions. Finally, contextual reflexivity entailed recognising her connection to the main recruitment partner with which she’s been collaborating as part of her doctoral studies, and how this positioning could potentially shape data interpretation.

## Results

### Participants characteristics

Participants (*n* = 19) ranged in age from 22 to 51 years (M = 33.88, SD = 8.01). The sample included a diverse mix of ethnicities, with participants from White (*n* = 10, 53%), Black/African/Caribbean/Black British (*n* = 6, 32%), Asian/Asian British (*n* = 2, 11%) and Mixed/Multiple ethnic groups (*n* = 1, 5%). Our sample included different professionals: most participants were SP link workers (*n* = 6, 32%) and youth workers (*n* = 3, 16%), with other roles including senior professionals (*n* = 3, 15%), creative practitioner (*n* = 1, 5%), occupational therapist (*n* = 1, 5%) and youth producer (*n* = 1, 5%). They mostly worked in Voluntary and Community Sector (VCS) organisations (*n* = 10, 53%) followed by arts organisations (*n* = 3, 16%), the NHS (*n* = 3, 16%), Local Authorities (*n* = 2, 11%) and the private sector (*n* = 1, 5%). On average, participants had 2.10 years of experience in their current role and 10.22 years of experience working with CYP.

### Research question 1: what are the active ingredients for SP when used to support the mental health of CYP?

We identified four themes: (1) SP offers a holistic approach that involves tailoring programmes to a young person’s needs, interests and their readiness, (2) SP offers young people an opportunity to exercise agency in defining their care pathway and engaging in their recovery journey, (3) The development of a professional but unpressured supportive relationship with a skilled adult is an important factor in SP, and (4) SP offers a safe space for young people to discuss their emotional needs and health.

#### SP offers a holistic approach that involves tailoring programmes to a young person’s needs and interests

Many participants described SP as person-centred and holistic, with care pathways tailored to the CYP’s interests and preferences. Understanding what is meaningful and motivating for CYP is essential for their engagement and benefit from SP, and some participants emphasised that this process requires time and effort.

*Because it’s all it’s a holistic approach*,* isn’t it? It’s all about one of us working together for the benefit of a young person*,* but us as professionals just have to be careful that we’re not*,* you know*,* overdoing it. Both the parent/guardian and the young person*,* that’s the only thing we need to be mindful of.* Female, 40 years, Youth Support and Development officer.

Sometimes, however, providing an activity that the CYP is interested in and will benefit from is not enough to ensure that they will engage with it. Some participants highlighted that the first session is key to building rapport and engagement with the CYP, explaining they use different strategies to support them attending in this first encounter.

*And then if they feel welcome*,* they will stay. So the first impression is quite*,* is quite important. And that’s the first impression that*,* at least is that*,* what the*,* the feedbacks that we have so and you can feel it anyway*. Male, 37 years, Creative practitioner.

#### SP offers young people an opportunity to exercise agency in defining their care pathway and engaging in their recovery journey

An important aspect of SP is its voluntary nature. From the outset, it is the CYP’s decision whether to take part in SP activities. Some participants identified that being ready to engage with this care pathway is a crucial factor in CYP recovery. At times, SP activities may not be suitable for CYP because, despite the support and encouragement provided, they are not ready to engage with activities.

*[…] because it depends where the person is at like in the cycle of readiness to change. If someone’s literally like*,* Oh I’ve been thinking about this for ages*,* I just needed some information. […] And for some people it’s just like even the idea of doing something it’s like so much.* Female, 37 years, Link Worker.

Once the CYP choses to join SP, they take an active role in defining their care pathway, supported by a professional. One participant defined it as the CYP taking ‘the driving seat’.

*So when I do the action plan*,* I don’t do it. I make the young person do it and see what are*,* so I ask what do you want to do? Where*,* what do you want to shape it? Is your mental health*,* is your physical health*,* whatever*,* it is*,* so how do we get there?* Female, age not disclosed, Youth worker.

Some participants explained that to follow the CYP’s lead, they would adopt a balanced attitude, encouraging participation and engagement with activities while respecting the limits and boundaries set by the CYP.

*So if they do disengage*,* we’ll obviously try and keep getting in touch with them but we’ll say if you want to say no then just say no*,* we’ll leave you alone.* Male, 33 years, Team Leader of SP programme.

For some participants, it is the CYP’s own perceptions and experiences of the engagement that will play a key role in their recovery journey. Many participants highlighted that the CYP must, in this process, recognise that it is within their power to improve and take ownership of their own recovery, exercising agency over it.

*Because ultimately*,* any improvement*,* any outcomes*,* are the young person’s to reach for and to own*,* no matter what interventions we might put into place.* Female, 46 years, Youth Service Senior Manager.

#### The development of a supportive relationship with a skilled adult is an important factor in SP

All participants reported on how they developed a supportive relationship with young people throughout their engagement. Support was provided in various ways, including maintaining motivation and engagement in activities, setting goals, managing expectations, and addressing any personal challenges that might arise from participation in SP activities.

*It’s something that they want*,* not me. So I can’t impose and say you shouldn’t do this this way. Because I think it’s better for you. No. You tell me what you think is better for you. And I can give you some options as well*,* I can complement that.* Female, age not disclosed, Youth worker.

Participants often spoke about the skills and training they bring into these relationships. Many emphasised the importance of listening and empathy, while some underscored relational abilities, the use of coaching techniques and adherence to youth work principles as further relevant skills.

*So yeah*,* those relational abilities to build relationships with people and assessing needs and being able to read between the lines and ask the right questions at the right time.* Male, 33 years, Team Leader SP programme.

A few participants also expressed their belief in SP and the emotional reward that comes with their role, which can relate to the personal efforts they reportedly add to the job.

*And so yeah*,* the core skills being really personal*,* personable but building relationships across the community is so key.* Female, 37 years, Link Worker.

#### SP offers a safe space for young people to discuss their emotional and health needs

Many participants described the importance of creating a safe space for young people engaging with SP. There are two components related to this. Firstly, many participants referred to a subjective component, as in building a non-judgemental space where CYP feels comfortable and everyone is treated equally, despite any particular mental health challenges they may be going through.

*And I feel like we still try and provide a really like non-judgmental*,* like supportive place*,* I feel like the content they would have had would still be very supportive*,* warm*,* non-judgmental*. Female, 37 years, Link Worker.

Many participants added an objective aspect, mainly developing appropriate safeguarding procedures. This also includes professionals receiving Disclosure and Barring Service (DBS) checks and safeguarding training.

*And we always talk about safeguarding at the start of the meetings*,* so I always explain that if you wanted to tell me anything or if you disclose anything that I think you might be a little bit in danger*,* or not well I do have to pass this on.* Female, 24 years, Link Worker.

### Research question 2: what contextual factors increase or decrease the effectiveness of SP with this population?

We identified four contextual factors commonly referenced in the data relevant to the second research question. They were (1) Supportive organisational environment, (2) Parental buy-in, (3) Public awareness, and (4) Barriers to access.

#### Supportive organisational environment

Many participants reported that the organisations they work at provide various forms of support for their delivery of SP activities. This includes ensuring that professionals have protected time for training and to acquire the necessary skills to perform their roles well. Training in therapy-related skills, motivational interviewing, child protection and safeguarding are among the relevant opportunities they accessed.

*So yeah*,* I’ve never had a situation where I felt like I didn’t know what to do and I didn’t feel supported. So no*,* definitely it’s a good environment.* Male, 37 years, Link Worker.

Some participants also referred to the benefits of having access to peer support and supervision, both for the SP link worker role and for other VCS based roles such as youth workers or mentors.

*I definitely have clients who I feel I’m stuck with*,* and that’s when supervision is so essential to talk through like the scenarios and the conversations.* Female, 37 years, Link Worker.

However, many participants highlighted that when organisations face difficulties such as funding and/or staffing constraints, this can affect how many CYP can be supported and the quality of this support.

*Organisations need to be*,* you know*,* funded right*,* you know*,* to take on referrals*,* we want to make sure that young people are in safe hands and legit situations*. Female, 40 years, Youth Support and Development.

#### Parental buy-in

Participants often referred to parents/carers as a potential challenge for CYP’s engagement, emphasising that securing their buy-in was fundamental for SP to be effective. The first aspect of this is obtaining their consent for the CYP to participate, which is mandatory for most services.

*But obviously*,* that’s the block*,* I need their consent in order to work with the young person though. That’s probably a challenge. And not being able to… Yeah*,* just regular contacts*. Female, 40 years, Youth Support and Development.

Secondly, some CYP may require greater support from their parents/carers, both logistically – such as transporting them to and from activities – and emotionally, by encouraging their child to remain involved in activities. However, parents/carers may lack the time or may not have sufficient information about SP to trust its effectiveness, which can limit how much their child benefits from activities.

*[…] but also maybe having mum and dad that might have*,* you know*,* more sporadic work*,* working commitments*,* that might mean that they’re not as able to support the young person*. Male, 26 years, Regional Project Manager.

Additionally, a few participants mentioned how a parent’s history of poor mental health can affect their ability to support their child’s attendance at SP activities.

*So parents are quite a big barrier either that they aren’t willing to do that bit of change for themselves to maybe get people to stuff they maybe aren’t able to consent due to their own childhood experiences and their own experience.* Male, 33 years, Team Leader SP programme.

#### Public awareness

Many participants commented on a lack of public awareness around SP which brings challenges for engaging both CYP and their parents/carers – if they do not know that the service is available in their communities, or do not understand what SP is, it is less likely they will access the activities offered.

*So how can they themselves know about it and get the benefits out of it?* Female, 28 years, Children and Young People’s Lead for Mental Health.

A few participants reported that the lack of awareness impact the organisations they work for who struggle to fill the spaces available in their SP programmes, adding pressure to the sustainability of such programmes.

*[…] I think that probably one of the challenges is actually getting people signed up and for people to know that we exist.* Female, 26 years, Occupational Therapist.

However, many participants detailed that when organisations exist within a collaborative community ecosystem that includes other VCS organisations, schools, families, mental health services and other statutory services, they can expand their reach.

*We work really closely with organisations like the SP scheme A*,* NHS*,* the police*,* we’re always working in referrals and for us*,* it helps to understand where that young person is coming from […]* Male, 32 years, Youth worker.

#### Barriers to access

All participants commented on barriers that affect how CYP access and experience SP. These barriers relate to three broad areas: (a) Geographical barriers, (b) Sociocultural barriers and (c) Health-related barriers.

##### Geographical barriers

Many participants reported that where the CYP lives influences how they experience SP. Distance from organisations, transport difficulties and the limited availability of activities in certain areas were the main geographical barriers mentioned.

*So it’s really*,* really very accessible even though it’s kind of 15 minutes’ drive away that just can’t get there because of the fact that they don’t have access to transport. So that’s a real frustration*. Male, 33 years, Team Leader SP programme.

One participant also mentioned that local gang affiliation can be an issue for some CYP. The so-called ‘post-code wars’ can compromise the safety of young people from certain areas when travelling to disputed areas and/or to areas dominated by rival gangs.

*There are also… reported by some young people within the Borough postcode related issues and things relating to local gang affiliation related matters in terms of travel and safety matters in terms of traveling from one geographical area to another.* Female, 46 years, Youth Service Senior Manager.

##### Sociocultural barriers

Many participants reported sociocultural barriers to accessing SP, including financial constraints. This suggests that some CYP lack the monetary resources to pay for certain activities and/or for transport to them.

*Sometimes access to sporting activities that come with a real high cost to be able to engage with them. So to join a football club you might have to spend 20*,* 30 quid on a pair of proper boots.* Male, 33 years, Team Leader SP programme.

Many participants also addressed how language can interfere in how SP is accessed and experienced, particularly for parents/carers for whom English is not their first language. This is a significant issue in areas with large immigrant populations. One participant also highlighted limited access for those who require sign language.

*We do work with Syrian refugee children*,* Afghani refugee children*,* and yeah*,* I think they miss out a little bit because yeah*,* we just can’t have a comprehensive enough offer to get across the language barrier as well as just culturally it’s so different.* Male, 37 years, Link Worker.

Finally, some participants addressed how stigma can affect young people’s engagement with SP activities. This stigma, often rooted in religious or cultural traditions or norms, can lead to family disapproval of CYPs’ participation in certain activities. Similarly, stigma may be associated with certain groups, such as young man or the LGBTQIA + community, which may prevent them from engaging in activities.

*And what I realized is*,* as well*,* religion plays a role in a lot of a lot of things at times. Well*,* so*,* obviously*,* music*,* certain religions it’s*,* you know*,* it’s against their religions*,* so we can’t engage young people that way. The young person might want to*,* but the parents no*,* we can’t*,* we can’t have you doing that.* Female, 40 years, Youth Support and Development.

##### Health-related barriers

Some participants reported on health-related barriers. These mainly related to mental health needs such as Special Education Needs and Disabilities (SEND) or severe mental illness. Participants reported that these prevent young people from participating in SP activities as professionals may be less prepared to support them. A few participants also mentioned that there was a limited offer of activities for young people with disabilities.

*[…] someone who has issues related to ASD and what they might feel like in a particular space and setting. So in this broad sense*,* ensuring that we’re able to make our spaces physically accessible*,* and in terms of sensory needs*,* as accessible as reasonably practicable.* Female, 46 years, Youth Service Senior Manager.

## Discussion

This study addressed two research questions key to the development of complex interventions.

### Active ingredients of SP for CYP’s mental health

First, we identified four themes that may correspond to the AIs of SP when used to support the mental health of CYP. These are components that should be present whenever the intervention is delivered to ensure that it achieves the intended change in outcomes. To our knowledge, this is the first study to focus on the AIs of SP for CYP’s mental health. AIs are often understood differently and used interchangeably with other concepts, such as behaviour change techniques (BCTs), mechanisms of change and others [21]. These themes are consistent with those identified in the adult SP literature. A realist review by Husk and colleagues [5] identified professionals’ skills as a key mechanism of SP’s effectiveness, alongside patients’ belief in treatment, presentation of referral, accessibility of activities, supported transit to the first session and change in patient condition or symptoms. Authors report that the referral must match patients’ needs and expectations, and the diversity of the format and delivery of activities influenced patient’s receptiveness. Our first theme (*SP offers a holistic approach that involves tailoring programmes to a young person’s needs and interests*) echoes this, highlighting the importance of the first encounter, whilst our second theme (*SP offers young people an opportunity to exercise agency in defining their care pathway and engaging in their recovery journey*) emphasises that readiness for treatment is not solely dependent on the type of intervention or the skills of the professionals, but also on how the CYP feel about being supported. Another review, focusing on identifying core components of SP link worker for adults with physical health and/or social needs, report on the background, training and skills needed by professionals involved with SP [32], which partially supports our third theme (*The development of a supportive relationship with a skilled adult is an important factor in SP*).

Only one review has looked directly at the AIs of SP for adults with mental health difficulties [13], which identified 22 BCTs in the literature, most of which were associated with social support. Also aligned with our third theme (*The development of a supportive relationship with a skilled adult is an important factor in SP*), this reinforces the importance of the relationship between the professional and the CYP. A meta-synthesis study focusing on the use of SP for loneliness [33] suggested that a possible mechanism underlying the effectiveness of SP is the provision of a safe and welcoming environment for individuals to practice social skills and build confidence, consistent with our fourth theme (*SP offers a safe space for young people to discuss their emotional needs and health*).

Previous research on SP for CYP’s mental health has looked at related but additional information such as barriers and facilitators, the results of which align with and complement ours. For example, as in our study, Hayes and colleagues [23] highlight the empathy and listening skills that professionals should bring when working with CYP. Together these findings emphasise the importance of a trusting, unpressured and horizontal relationship between professionals and CYP.

Finally, our study identified CYP’s agency as a key active ingredient for SP focused on CYP mental health. This speaks to previous research in adults linking SP with empowering individuals to take responsibility for their own health [6] and reinforces that CYP should play an active role in defining and engaging with their care pathway. Developing agency is an important milestone in this age range, as CYP self-identity changes, acquiring responsibility about their own future is crucial for their independence and resilience [34].

### Contextual factors

We identified four contextual factors which may also influence the effectiveness of SP for CYP with mental health difficulties. Understanding how complex interventions interact with the context in which they are implemented helps to address issues of implementation and transferability in different settings [24].

Contextual factors have been explored to some extent in the adult and CYP SP literature. Previous research has found that ‘supporting the supporter’ [6] through training, supervision and peer support has a fundamental role in intervention delivery [6, 35], which aligns with our first theme (*Supportive organisational environment*). The second and third themes identified by this study have not been directly addressed in the SP literature: *Parental buy-in* and *Public Awareness*. Whilst Hayes and colleagues [23] mention the difficulties that professionals face when engaging with parents/carers, our study identified that SP cannot be effective without their support. A supportive family environment is often linked to good mental health in CYP [36]. For SP, this translates as support beyond consent, including active participation of parents/guardians in the CYP’s engagement with activities. Previous studies do not mention public awareness as a relevant factor in SP, perhaps because participants are usually those directly involved with delivering or taking part in SP.

In a realist review, Calderón-Larrañaga and colleagues highlight that accessing prescribed activities depends on a series of factors, including resources, cost, timing, location, variety and social and cultural appropriateness [35]. This broadly relates to our fourth theme (*Barriers to access*) and the geographical and cultural barriers that may hinder CYP’s access to support. However, there is no mention to health-related barriers, which should be considered for ensuring that interventions are equally accessible to people with varied mental health needs and disabilities. Regarding SP for CYP’s mental health, Hayes and colleagues [23] mention of population limitations and the need for funding to support CYP engagement in activities corroborates our fourth theme (*Barriers to access*).

### Limitations

Our study makes a novel contribution to the literature by exploring AI and contextual factors in the delivery of SP to support CYP’s mental health. However, some limitations should be noted. Although our participant pool was diverse, greater representation from minority backgrounds is encouraged in future research. Approximately half of the participants were recruited from the same SP programme, which may have influenced the findings by reflecting the specific characteristics of that programme. To mitigate this, we recruited participants from other sites.

### Implications for practice and future research

**Fig. 1:**
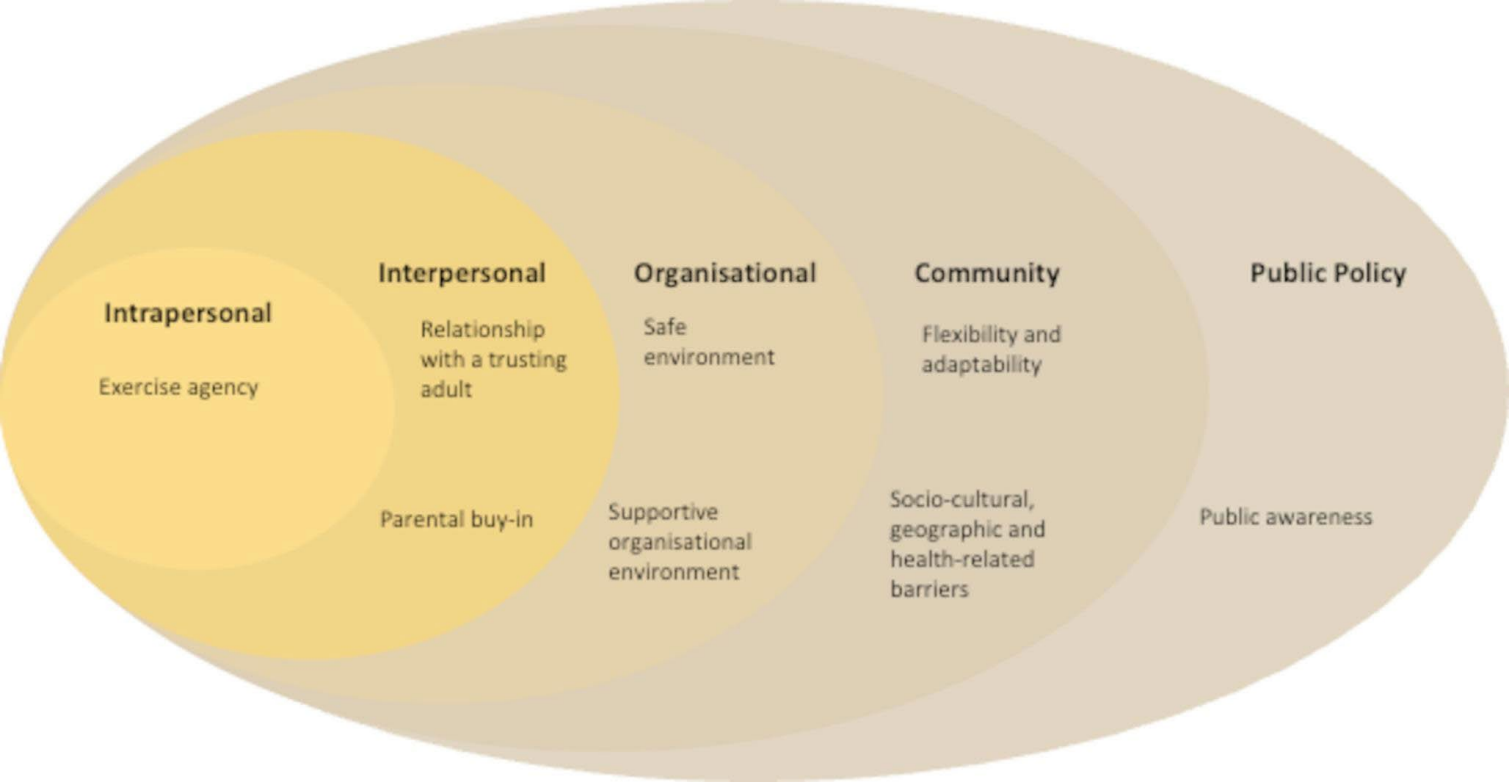
A socio-ecological framework for active ingredients and contextual factors for SP for CYP’s mental health

These findings provide a range of AIs and contextual factors for different stakeholders to consider when designing and delivering SP for CYP’s mental health. These findings can be situated within a socio-ecological framework, which has previously helped to understand different interventions such as parent-focused adolescent mental health prevention programmes [37] and training and mentoring for black youth [38]. Figure 1 illustrates how identified AIs and contextual factors align with the socio-ecological framework.

This framework looks at individuals, their interactions with the environment and how they influence each other [39]. Presenting the findings of our study in this way can help to inform the implementation of SP for CYP’s mental health by highlighting the levels at which AIs and contextual factors are situated, identifying key responsibilities and tackling challenges while also leveraging opportunities for impactful change.

Future research could build on this framework to explore the barriers and facilitators associated with the implementation of these mechanisms and factors in different settings and populations, maximising intervention effectiveness. In this way, the framework highlights a potential link between the lack of public awareness of SP and the lack of structured policy and guidance for its implementation, particularly in the context of targeting CYP’s mental health [23]. Additionally, defining the AIs and contextual factors of SP for CYP’s mental health may further consolidate the practice and consequently contribute to its wider uptake. It is also advisable to explore how these AIs and contextual factors relate to the outcomes identified in our study to contribute to an understanding of what works, for whom and in what circumstances [40]. Finally, further research with CYP involved in SP programmes and their families is paramount to enable exploration of their experiences and perceptions of SP and its benefits.

## Electronic supplementary material

Below is the link to the electronic supplementary material.

Supplementary Material 1

## Data Availability

No datasets were generated or analysed during the current study.
