# Supplementary material for: Identifying the active ingredients and contextual factors of social prescribing when used to support the mental health of children and young people: a qualitative study
Source: Eur Child Adolesc Psychiatry. 2025 May 17;34(10):3249–59. doi: 10.1007/s00787-025-02734-5 (PMC12592259; doi:10.1007/s00787-025-02734-5)
Supplement: Supplementary file 1 — Supplementary Material 1 [file 787_2025_2734_MOESM1_ESM.docx]

**Topic Guide**

**Interview with Professionals delivering SP for CYP with MH difficulties**

*Thank participants for their availability, introduce yourself and start recording:*

**Opening questions**

1. Please, tell us about your experience as a professional delivering SP for children and young people.

- How many young people have you worked with as part of the scheme?
- What does your organisation usually offer to these young people?
- How frequently do you see each young person during the time they are with your organisation?
- How long is your engagement with participants, on average? (months, number of sessions)

1. How is a person “discharged” from your care and support? What leads to the end of the engagement? Is there a standard amount of time or does it change per case?

- How long it usually takes for you to feel the young person has improved? Does it change per case or it’s usually the same average for all? Were there times where you couldn’t notice any improvement?
- Are these young people different from others you work with as part of your role in the organisation? How?

**Benefits from Social Prescribing**

1. In your experience, how helpful has social prescribing been for young people?

- Can you name some of the benefits?
- And some aspects that might not have been so useful for them?

1. What aspects do you think young people find most enjoyable and helpful? Why?
2. Why is it important to consider young people’s interests when suggesting activities in social prescribing? Does it make a difference?
3. Do you often get good feedback from families? What kind of things do they say? And young people, do they share any feedback?
4. If you were advising friends/family, would you recommend social prescribing? Why?

**Challenges**

1. What are the challenges for young people in access to social prescribing scheme?
   1. Are some groups more disadvantaged by others in terms of access? Are there any barriers connected to a specific group? (age range, ethnical background, etc.)
   2. How would you improve access to the social prescribing scheme?
2. Have any of the young people you worked with within the social prescribing scheme dropped out before the completion of the assigned time? Why?
3. Is there a particular group who seem to benefit more from social prescribing? (Gender, marginalisation, ethnical background, etc.) Why?

1. What are the outcomes the organisation expects from a child or young person joining the social prescribing scheme?

- How are these outcomes assessed? (e.g. validated scales/measures, exit interviews, feedback questionnaires, etc…)
- What are your views on this assessment? Do you think the measures work well?
- Is there anything you would change about it?

**Experience working with SPCYP**

1. Can you tell us what are the difficulties in conducting your work?

1. During your time working for this social prescribing scheme, have you ever experienced something unsafe or distressing happening? Can you tell us more about it?

- How did you manage it?
- Did you feel you were supported/well-equipped to manage that?
- What kind of support did you receive from your organisation? And from the social prescribing scheme partners (NHS, city council, etc.)?

1. Did you get any training before starting as a social prescribing worker? Which ones? Were you ever taught about any theories or models about social prescrihing?

1. Looking ahead, what sort of training would help you to deliver your work better?

**Closing questions**

1. Do you have anything else to add to what has been discussed today: any further comments about your experiences of working with young people in a social prescribing scheme?
2. Do you have any questions about this study?

*Stop recording and thank participant for their contribution*
